# Assessing receptivity to malaria using case surveillance and forest data in a near-elimination setting in northeast Thailand

**DOI:** 10.1186/s12936-024-05044-4

**Published:** 2024-07-30

**Authors:** Rebecca Walshe, Kulchada Pongsoipetch, Suwanna Mukem, Tanong Kamsri, Navarat Singkham, Prayuth Sudathip, Suravadee Kitchakarn, Rapeephan Rattanawongnara Maude, Richard James Maude

**Affiliations:** 1 Mahidol Oxford Tropical Medicine Research Unit, Faculty of Tropical Medicine, Mahidol University, Bangkok, Thailand; 2 Faculty of Medicine, Ramathibodi Hospital, Mahidol University, Bangkok, Thailand; 3 Phibun Mangsahan Hospital, Phibun, Ubon Ratchathani Thailand; 4 Provincial Health Office, Ubon Ratchathani, Thailand; 5 Buntharik Hospital, Buntharik, Ubon Ratchathani Thailand; 6 Division of Vector Borne Diseases, Department of Disease Control, Tiwanond Road, Nonthaburi, 11000 Thailand; 7 Centre for Tropical Medicine and Global Health, Nuffield Department of Medicine, University of Oxford, Oxford, UK; 8 The Open University, Milton Keynes, UK

**Keywords:** Receptivity, Malaria, Transmission, Indigenous, Surveillance, Forest

## Abstract

**Background:**

Thailand aimed to eliminate malaria by 2024, and as such is planning for future prevention of re-establishment in malaria free provinces. Understanding the receptivity of local areas to malaria allows the appropriate targeting of interventions. Current approaches to assessing receptivity involve collecting entomological data. Forest coverage is known to be associated with malaria risk, as an environment conducive to both vector breeding and high-risk human behaviours.

**Methods:**

Geolocated, anonymized, individual-level surveillance data from 2011 to 2021 from the Thai Division of Vector-Borne Disease (DVBD) was used to calculate incidence and estimated R_c_ at village level. Forest cover was calculated using raster maps of tree crown cover density and year of forest loss from the publicly available Hansen dataset. Incidence and forest cover were compared graphically and using Spearman’s rho. The current foci classification system was applied to data from the last 5 years (2017–2021) and forest cover for 2021 compared between the classifications. A simple risk score was developed to identify villages with high receptivity.

**Results:**

There was a non-linear decrease in annual cases by 96.6% (1061 to 36) across the two provinces from 2011 to 2021. Indigenous Annual Parasite Index (API) and approximated R_c_ were higher in villages in highly forested subdistricts, and with higher forest cover within 5 km. Forest cover was also higher in malaria foci which consistently reported malaria cases each year than those which did not. An R_c_ > 1 was only reported in villages in subdistricts with > 25% forest cover. When applying a simple risk score using forest cover and recent case history, the classifications were comparable to those of the risk stratification system currently used by the DVBD.

**Conclusions:**

There was a positive association between forest coverage around a village and indigenous malaria cases. Most local transmission was observed in the heavily forested subdistricts on the international borders with Laos and Cambodia, which are where the most receptive villages are located. These areas are at greater risk of importation of malaria due to population mobility and forest-going activities. Combining forest cover and recent case surveillance data with measures of vulnerability may be useful for prediction of malaria recurrence risk.

## Background

As countries approach the elimination phase of malaria control, universal interventions yield diminishing returns. The identification of local areas which are receptive to malaria allows the rationalization of resources to focus efforts on reducing the risk of re-emergence in these areas. In 2016, Thailand, in the Greater Mekong Subregion (GMS), set the goal to eliminate malaria by 2024, where elimination is defined as “the reduction to zero of local, or indigenous, malaria incidence” [1]. As such, Thailand is doing prevention of re-establishment (POR) planning for malaria free provinces [2]. The World Health Organization (WHO) recommends that malaria endemic countries identify areas which are receptive to malaria, including where transmission has been curtailed by current interventions [3]; however, there is no established method to measure this and no “gold standard” available to evaluate assessment methods [4].

The WHO defines receptivity as “(The) degree to which an ecosystem in a given area at a given time allows for the transmission of *Plasmodium* spp. from a human through a vector mosquito to another human” [5]. This concept reflects the vectorial capacity of the mosquito, susceptibility of the human population to malaria infection and the strength of the health system, including malaria interventions. It is therefore influenced by local ecological and climatic factors.

A challenge facing countries aiming to use receptivity mapping to inform future malaria control strategies is the lack of standardized methods to achieve this [4]. Approaches are therefore diverse and depend on the data available [4]. Quantitative methods largely use an estimated form of the reproduction number (R): commonly R_c_ (R where control interventions are in place) and R_e_ (R accounting for acquired immunity) [6–9]. Where a strong surveillance system is not in place, vectoral capacity approximated as biting rate [10, 11] and historical or approximated parasite rate [12, 13] has been used*.* Other approaches include the use of environmental data [14] and entomological surveys [4, 10, 15]. The absence of malaria vectors in an area can be used to infer that it is not receptive [3]. However, this relies on collecting and identifying an adequate sample of potential vectors and is susceptible to sampling error. In most cases, the best approach is that which uses the best available relevant data.

The relationship between forests and malaria is complex and has not been fully characterized throughout the GMS. Forested areas are conducive to malaria transmission due to a combination of environmental factors including vegetation cover, temperature, rainfall, humidity and lack of infrastructure [16]. Attempts to define the spatiotemporal relationship between forest cover changes and malaria in the Amazon rainforest have included the malaria frontier hypothesis [17] and more recently the deforestation-malaria hypotheses [16, 18]. However, there are different ecological and human processes in the GMS: crucially, people more often engage in intermittent forest-going activity for work than to establish permanent settlements in the forest [19]. The predominant vectors in the GMS (*Anopheles minimus* and *Anopheles dirus*) breed in and around forests. One study using data from Lao PDR and the Hansen forest dataset found that there was an increased malaria burden in the 2 years after deforestation within a 10 to 30 km radius of a village, followed by a subsequent reduction [20]. This was more marked for *Plasmodium falciparum*, and there was no significant relationship with deforestation within a 10 km radius. In Thailand, active malaria foci are more likely to be found in areas with tropical forest or plantations, and disturbance of the forest [2]. The vulnerability of an area, or the risk of parasite importation via population movement, is also higher in the forested border regions, where there are often migrant workers and forest-goers [2]. This mix of environmental and behavioural factors places forested regions at a higher risk of re-emergence of local transmission where malaria has previously been cleared [16].

**Fig. 1:**
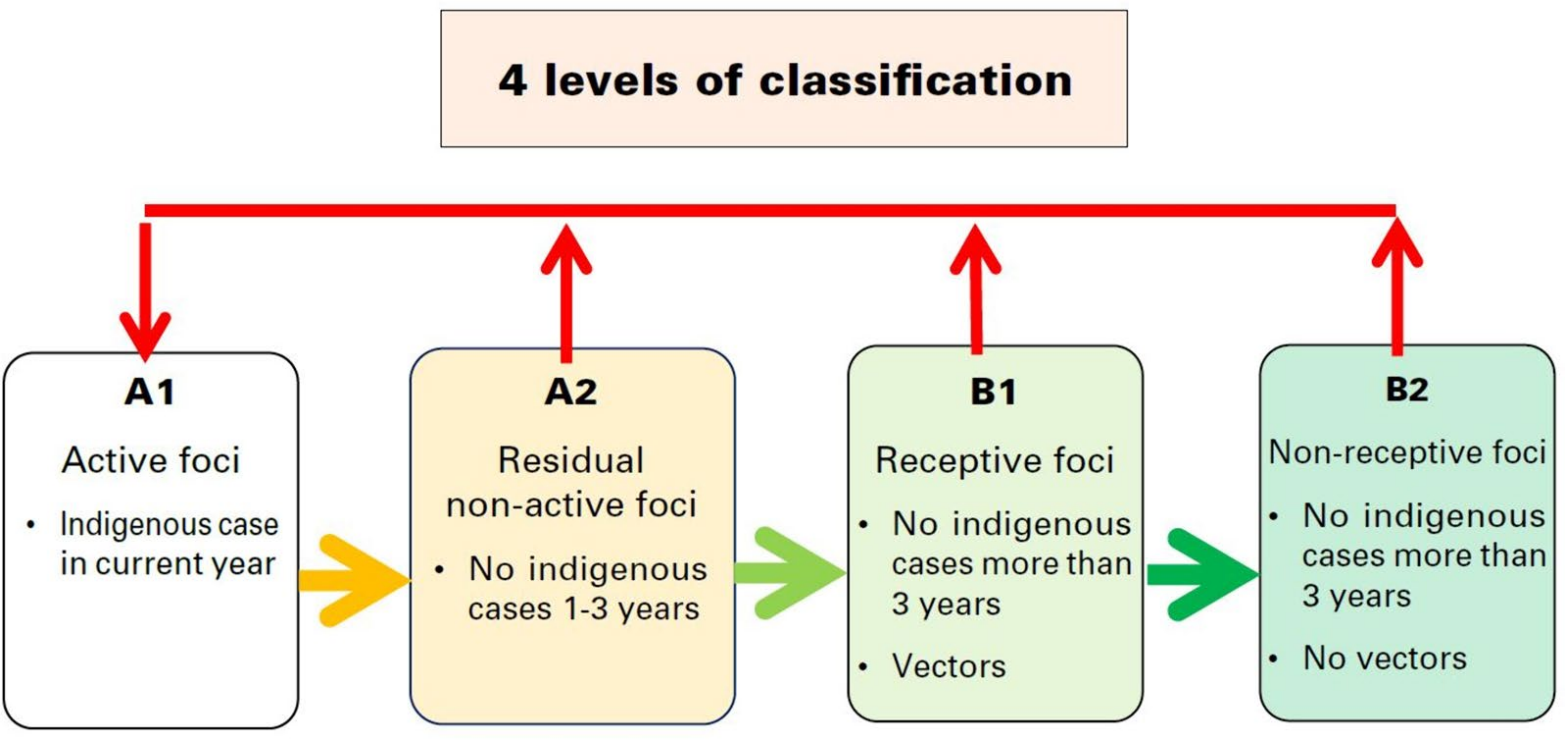
Current risk classification system applied to individual villages in Thailand

Foci management is resource-intensive, so it is important to accurately identify (a) local malaria transmission and (b) receptive areas*.* In Thailand at present, malaria risk stratification is based on case surveillance data and the presence of vectors [1]. The operational definition of an indigenous case in this setting is a malaria case acquired within the village of residence, which is determined by case investigation [1]. Since 2017, villages in Thailand have been stratified into four foci types [1, 21, 22] per Fig. 1. A1 foci, defined as “villages with reported indigenous cases in the current fiscal year”, receive passive as well as biannual active case detection; supervised radical cure; case investigation; insecticide-treated nets (ITNs) and indoor residual spraying (IRS); and entomological studies if cases persist for over 4 weeks [1, 21]. If they subsequently have no cases the following year, they are classified as A2 foci for the next 3 years and receive less frequent active case detection. B1 foci are villages with no indigenous transmission in the last 3 years that are still considered receptive to malaria due to the presence of vectors. Vectors have not been found in B2 villages, and so they are not considered to be receptive. The main intervention difference between B1 and B2 villages is the delivery of malaria education for residents whose occupation involves going into the forest at night time [1]. Such targeted educational efforts and other interventions, including on the use of ITNs, can have mixed uptake [19]. Thailand has also been implementing the 1-3-7 surveillance strategy since 2016 [21, 22]. This requires case notification within 1 day, investigation within 3 days, and foci investigation within 7 days of each confirmed malaria case. Likely in response to this intensive elimination strategy, malaria incidence is decreasing and adherence to the surveillance programme is improving [22].

When assessing the receptivity of individual villages to malaria, it is useful to consider malaria cases identified as having been transmitted within the village: if a person is infected by an anopheles mosquito which in turn has been infected from a nearby human blood meal, it follows that the local environment is receptive to malaria transmission. The current risk stratification system recommended by the Thai Division of Vector-Borne Disease (DVBD) involves the most active interventions in villages with recent reported cases, and the collection and identification of vectors in mosquito traps in villages which have not reported indigenous cases within the last 3 years (Fig. 1) [1]. However, this approach can be labour-intensive, prone to sampling error, and requires entomological training to accurately identify the mosquito species [4, 23, 24]. Based on a large dataset of malaria surveillance records and publicly available satellite forest data, the aim was to quantify the relationship between local forest cover and malaria transmission in order to help to inform an objective and resource-efficient way of stratifying individual village receptivity and malaria risk as Thailand reaches the POR phase, which the DVBD is currently developing [2].

## Methods

### Study setting

The study areas were Si Sa Ket and Ubon Ratchathani provinces in Northeast Thailand, which are subdivided into 22 and 31 districts, respectively. Ubon Ratchathani borders Lao PDR to the east, and Cambodia to the south. Si Sa Ket is located to the west of Ubon Ratchathani and shares its forested southern border with Cambodia.

### Village population and location

**Fig. 2:**
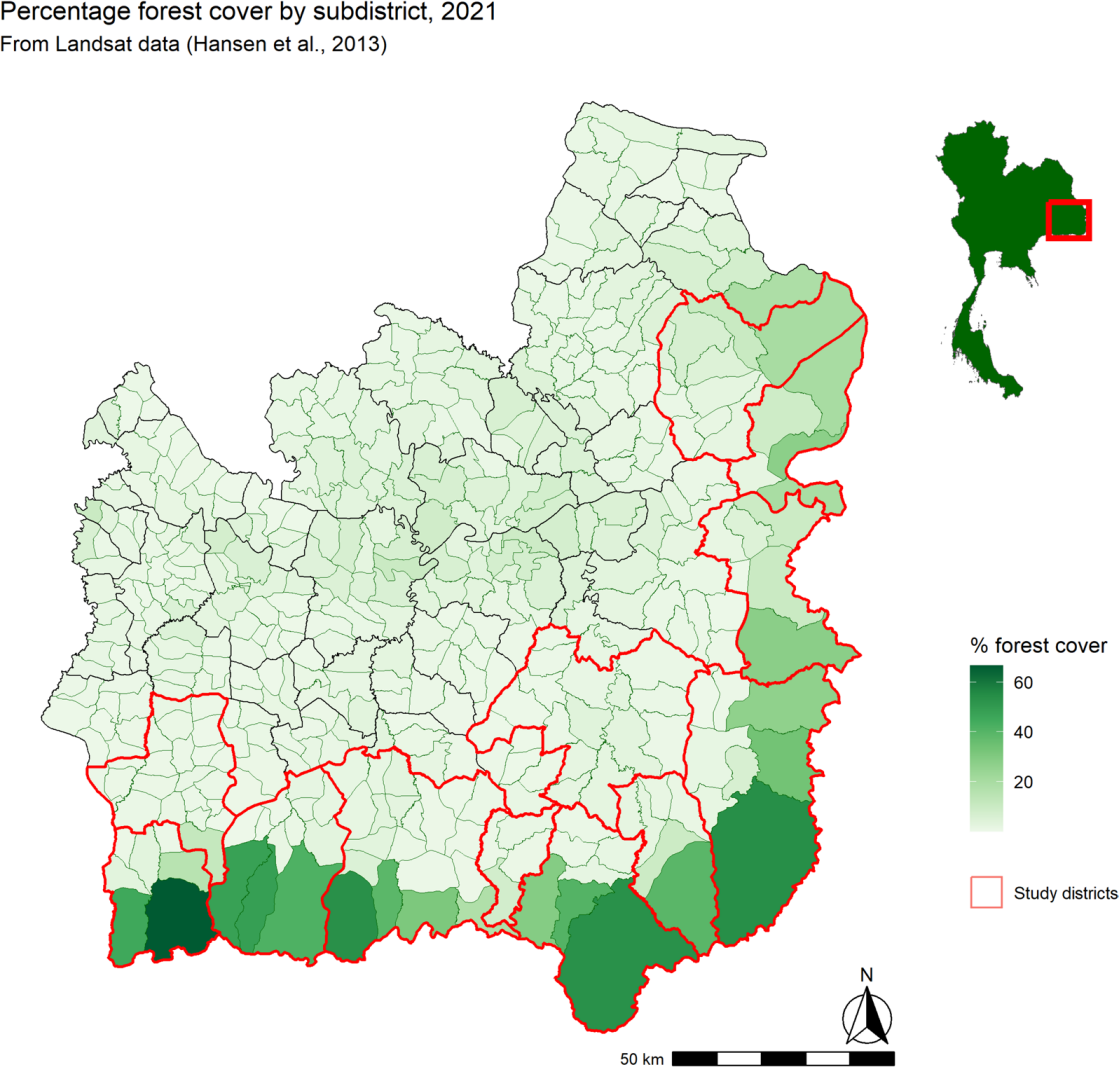
Percentage forest cover by subdistrict in 2021. Red lines outline the study districts

All villages in the 12 districts contributing 95% of all malaria cases in their province were included in this analysis (Fig. 2; outlined in red). 1640 villages were geolocated in the 12 districts, representing 29.79% (786 villages) of all villages in Si Sa Ket and 31.58% (854 villages) of those in Ubon Ratchathani. Three sources were used: (1) Manual validation of GPS co-ordinates from the DVBD surveillance database (2) GPS co-ordinates manually collected by MORU field staff covering all villages in four districts and (3) Street View in Google Maps. The co-ordinates were added to a list of annual population counts of villages in the study districts, published online by the Administration and Registration Technology Development Division, Bureau of Registration Administration, Department of Provincial Administration (DOPA), Ministry of Interior [25].

### Malaria case data

Individual anonymized records of malaria cases in Si Sa Ket and Ubon Ratchathani from 2011 to 2021 were provided by the Thai DVBD. The majority of malaria cases in Thailand are diagnosed by passive case detection at malaria posts, health-promoting hospitals, and district hospitals. A positive malaria blood smear or rapid diagnostic test (RDT) result is reported by government health workers [1] to the Thai DVBD.

From 2011 to 2021, there were 7942 cases of malaria in Si Sa Ket and 16,283 cases in Ubon Ratchathani in the raw dataset. 7825 cases (98.53%) in Si Sa Ket and 15,745 cases (96.69%) in Ubon Ratchathani were included in the full analysis following the exclusion of cases with incomplete village data. Indigenous malaria transmission was considered at two levels: within the village of residence, and within the subdistrict of residence. Cases were therefore respectively aggregated by blood draw year and village, or subdistrict, of likely infection.

The approximated R number under control (R_c_) was calculated as the ratio of indigenous (to the village) and imported cases per year, per village [26]. API was calculated as the annual number of cases per 1000 of the population.

### Forest cover

Forest cover was calculated at the subdistrict and village levels using the 2021 update of the publicly available Hansen forest data [27] (Global Forest Change). The Hansen  dataset consists of layers produced from Landsat data using decision tree classifiers, at a 30 m spatial resolution. Tree cover is defined as “all vegetation taller than 5 m in height” [27].

Forest cover was calculated using raster maps of tree crown cover density in the year 2000 by 30 m pixel; and of year (2001–2021) of forest loss in those pixels from the relevant geographic area (20N 100E). The rasters were cropped to the administrative boundaries of Si Sa Ket and Ubon Ratchathani, with a surrounding buffer. For each subdistrict, the mean percentage tree crown cover density was calculated across all pixels within the subdistrict administrative boundaries in the year 2000. If marked as deforested in the forest loss dataset in a year between 2001 and 2021, the pixel value was subsequently set to zero. The mean cover was then re-calculated for each subdistrict, accounting for these deforested pixels, to give an approximation of forest cover in the year 2022, per the method described by Rerolle et al*.* [20]. Forest cover loss was calculated as the difference between the cover in 2011 and 2021.

Using QGIS [28], the percentage forest cover surrounding each village was calculated by producing a circular buffer zone around each village point location and calculating the zonal statistics, including mean percentage forest cover, for pixels within that buffer zone. This was calculated within a 1 km, 2 km and 5 km radius of each village, to account for potential variation in the flight distance of local mosquito species, the size of different villages and patterns of frequent human travel around their identifying point.

A nearest neighbour analysis was performed to identify the minimum distance to a forested pixel for each village co-ordinate using the distance to nearest hub tool in QGIS. Due to the small 30 m^2^ pixel size for forest data, a minimum percentage forest cover was set as 25% tree crown density per Hansen et al*.* [27].

### Analysis

Correlations between percentage forest cover metrics, estimated R_c_ and API were examined graphically using R [29], and quantified with Spearman’s rank using the cor.test() function in R. Spearman’s rank was used due to the non-normal distribution of the data. Due to the clustering of low forest cover and API values, the natural log of both was taken when comparing API and the percentage forest cover within a 1 km, 2 km and 5 km radius of each village point.

A sub-analysis was performed using only malaria data from 2017 to 2021, when the 1-3-7 reporting system and new malaria elimination strategy were introduced. The numbers of years in which villages reported malaria cases were plotted against indigenous API and forest cover. The villages were classified into A1, A2 and B per Fig. 1 for the years 2020 and 2021. Then, based on whether they subsequently reported indigenous malaria cases, they were categorized into (1) A1/A2 foci with subsequent indigenous cases (2) A1/A2 foci without subsequent cases and (3) No cases in past 5 years. There were no villages in the B classification (which reported no cases in 2017–2019) which subsequently reported an indigenous case in 2020 or 2021. Forest metrics were then compared between categories.

A new village malaria risk classification tool was developed based on the above data. This included the number of years in the past 5 with reported indigenous malaria cases, and percentage subdistrict forest cover. Villages were categorized into high risk; medium risk; low risk; and not receptive. These approximate roughly to the A1; A2; B1; and B2 classifications currently used by the DVBD. Maps of the classified villages were created and compared visually.

## Results

### API

**Fig. 3:**
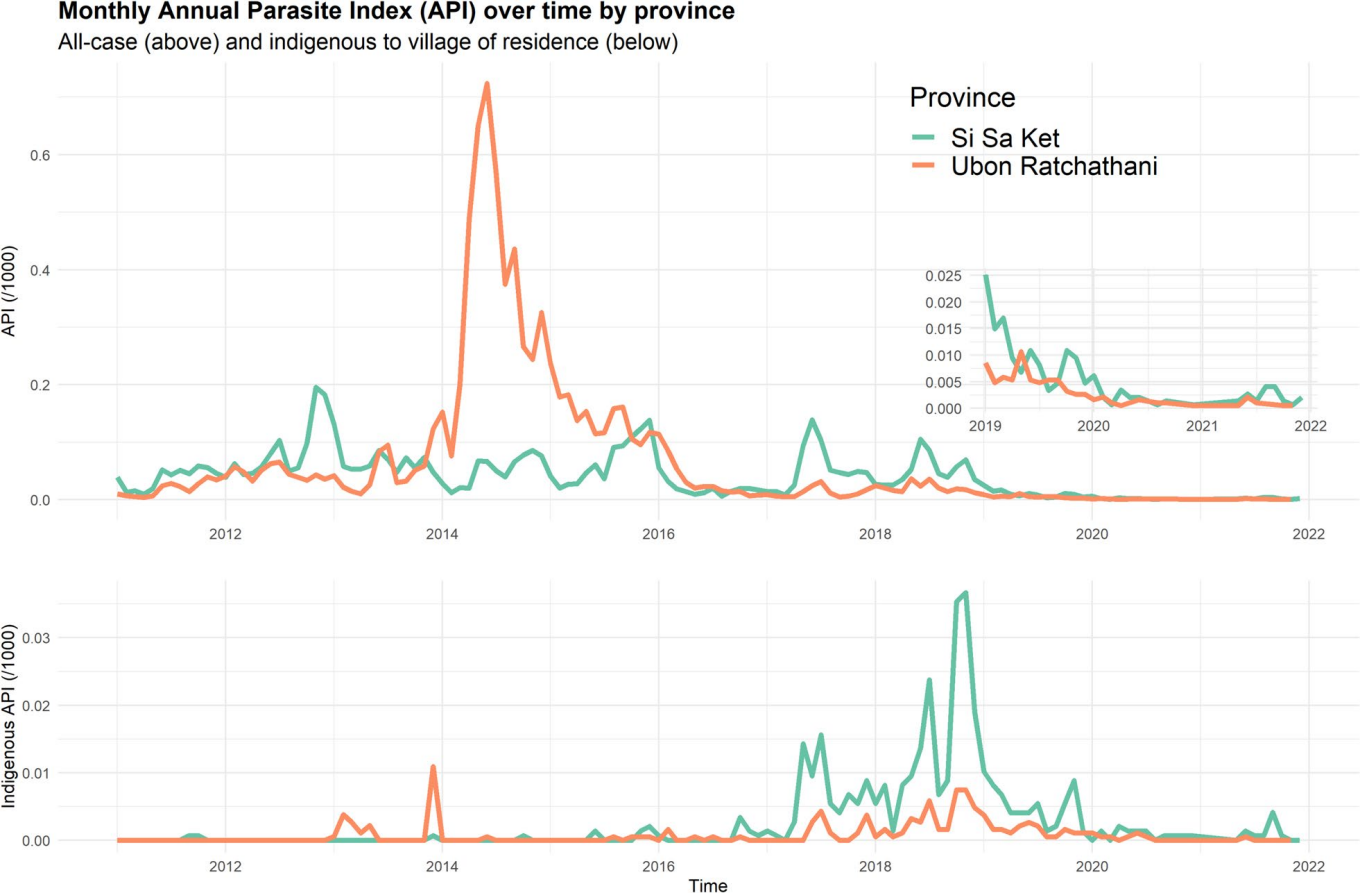
Annual Parasite Index (API), calculated as the number of cases per 1000 population, by province

From 2011 to 2021, there has been an overall negative trend in all-case and indigenous API in both provinces (Fig. 3). All-case API was higher in Si Sa Ket in most years, except between 2014 and 2016. There was a cross-border outbreak between Laos and southeast Ubon Ratchathani (Ubon) in 2014 [23], resulting in a large peak of cases mainly concentrated in the districts adjacent to the Laotian border. The 2014 peak in cases indigenous to the village of residence was relatively small, suggesting that the outbreak consisted of largely imported or unclassified malaria cases. Indigenous API was otherwise higher in Si Sa Ket than Ubon, with an extended peak in API between 2017 and 2020.

### Forest cover

The subdistricts with the highest percentage of land area as forest cover were those on the southern borders in both provinces (Fig. 2). Dong Rak in Si Sa Ket had 66.5% forest cover; Huai Kha in Ubon Ratchathani 53.75%; and Lalai in Si Sa Ket 53.5%. The lowest forest cover percentage was in Chot Muang and Ta Ut subdistricts in Si Sa Ket (0.02%). The 19 least forested subdistricts were in Si Sa Ket, whereas the most forested subdistricts were found in both Ubon Ratchathani and Si Sa Ket*.*

### Forest cover and malaria cases

**Fig. 4:**
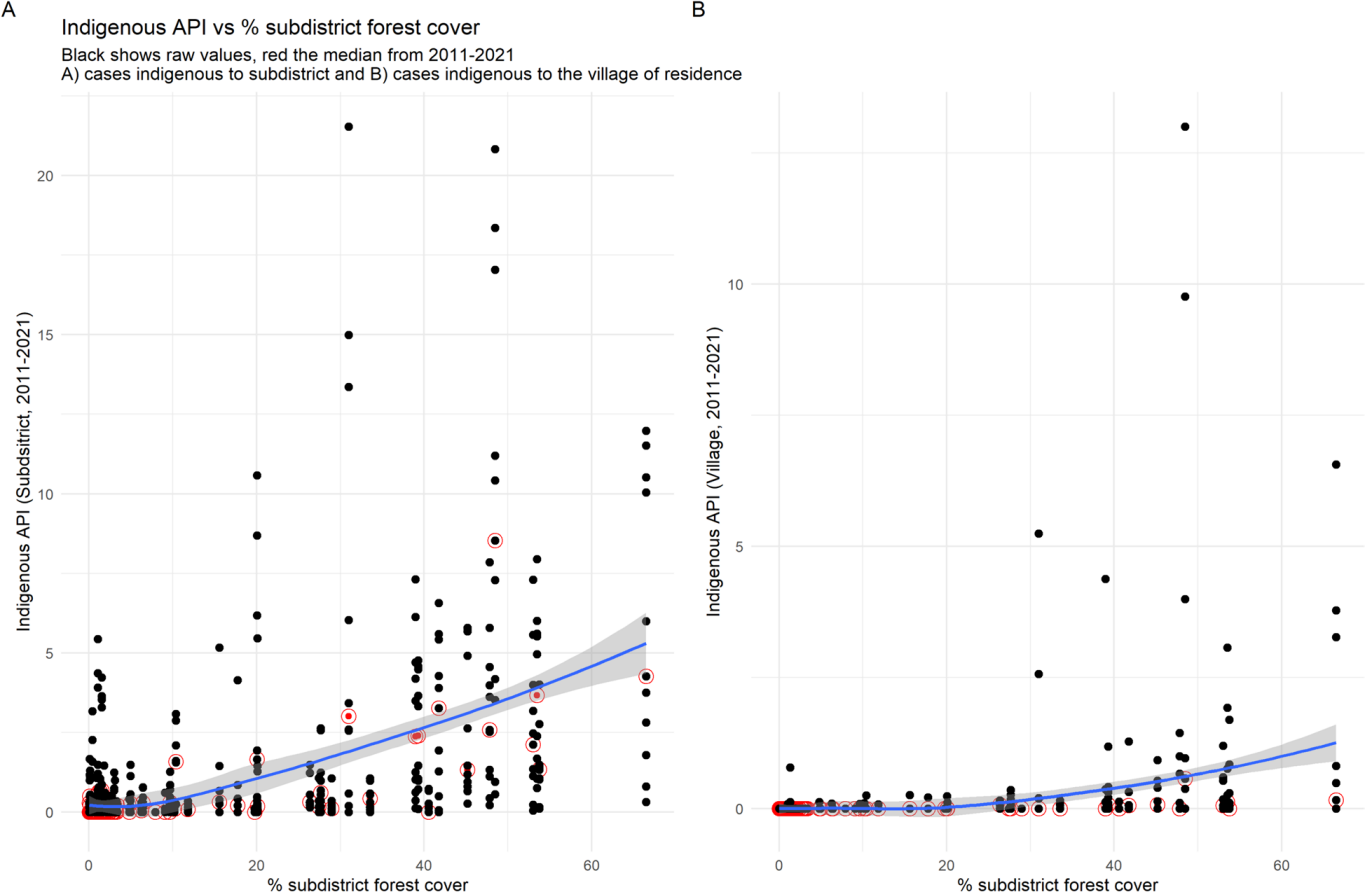
Indigenous API vs subdistrict forest cover 2011–2021. The trendline is fitted to raw values by Loess. API: Annual Parasite Index

When subdistrict percentage forest cover was plotted against API for indigenous cases transmitted within the village or subdistrict of residence (Fig. 4A), there was initially a relatively flat trend below 10% cover, beyond which there was a moderately positive relationship (Table 1: Spearman’s rho = 0.523). A similar pattern with a positive trend beyond 25% forest cover was observed when defining indigenous transmission as within the reporting village only (Fig. 4B; rho = 0.458). The correlation between subdistrict forest cover and all-case API was also moderately strong (rho = 0.456).

**Table 1:** Correlation of forest cover within different areas around each village with malaria API and Rc

	% forest cover within the subdistrict	% forest cover within 1 km radius	% forest cover within 2 km radius	% forest cover within 5 km radius	Distance to nearest > 25% forested pixel
All case API	0.456, p < 0.01	0.266, p < 0.01	0.294, p < 0.01	0.388, p < 0.01	− 0.140, p < 0.01
Indigenous API (Subdistrict)	0.523, p < 0.01				
Indigenous API (Village)	0.458, p < 0.01	0.147, p < 0.01	0.166, p < 0.01	0.235, p < 0.01	− 0.08, p < 0.01
Rc (Ratio of village indigenous:imported cases)	0.228, p < 0.01	0.159, p < 0.01	0.186, p < 0.01	0.271, p < 0.01	− 0.05, p = 0.01

There were weakly positive relationships between API (all case) and forest cover within a 1 km (rho = 0.266); 2 km (rho = 0.294); and 5 km (rho = 0.388) radius of the village (Table 1).

**Fig. 5:**
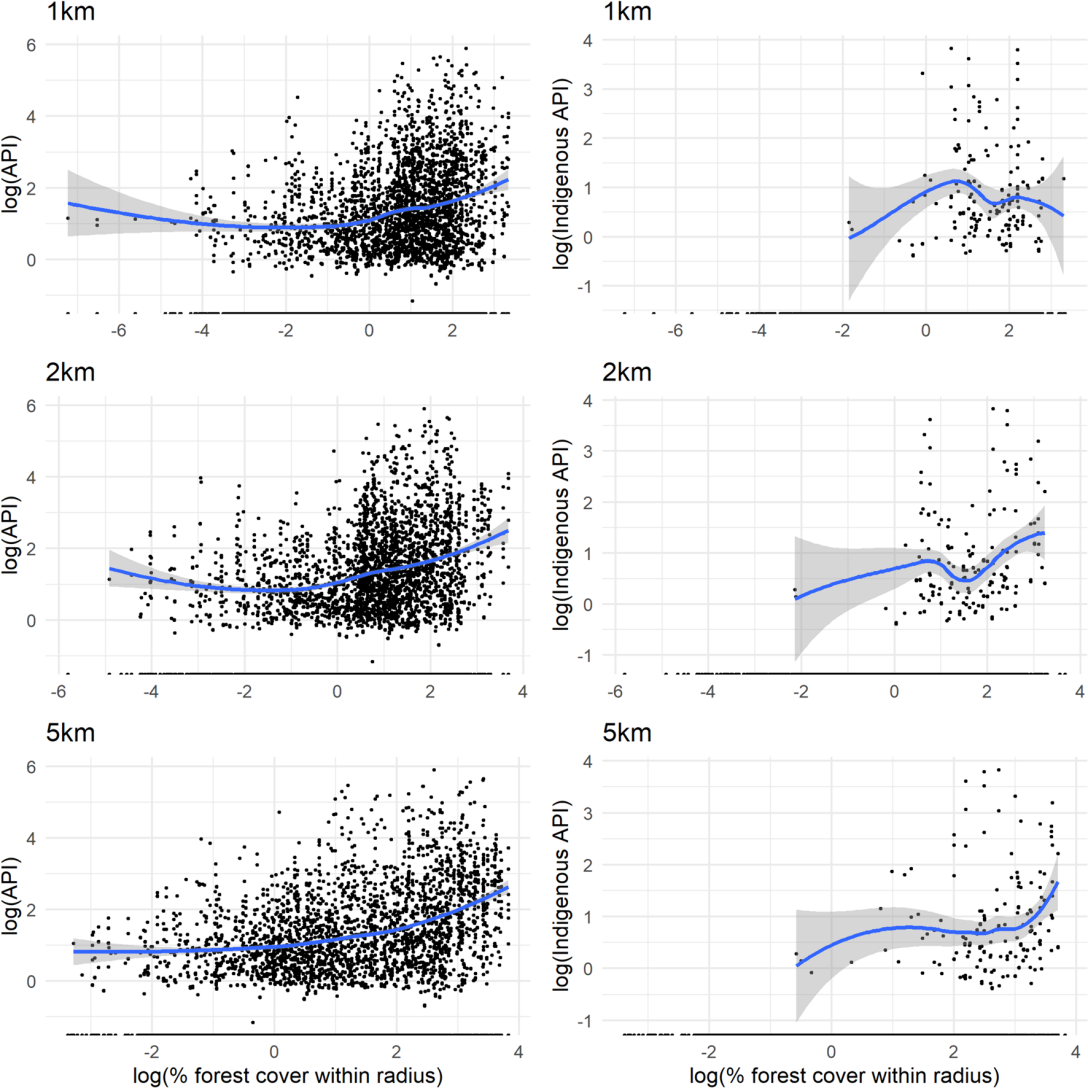
Log–log curves of all-case (right) and village indigenous (left) API vs percentage forest cover. Within a 1 km, 2 km, and 5 km radius of the village. API: Annual Parasite Index

When comparing API with forest cover within a 1, 2, or 5 km radius of the village (Fig. 5), there was a linear relationship above 1% forest cover (log[forest cover] = 0) within 1 km and 2 km; and an increasing gradient above 7.4% forest cover (log[forest cover] = 2) within 5 km. There were fewer non-zero data points for village indigenous cases, for which the relationship with forest cover was less clear. There was a mostly negative relationship between 2 and 5% forest cover (log[forest cover] = 0.8 to 1.6) within 1 km and between 2.7 and 4.5% (log[forest cover] = 1 to 1.5) within 2 km. Within a 5 km radius, there was a strong relationship between percentage forest cover and indigenous API above 20% cover (log[forest cover] = 3), but overall a low correlation (rho = 0.235).

**Fig. 6:**
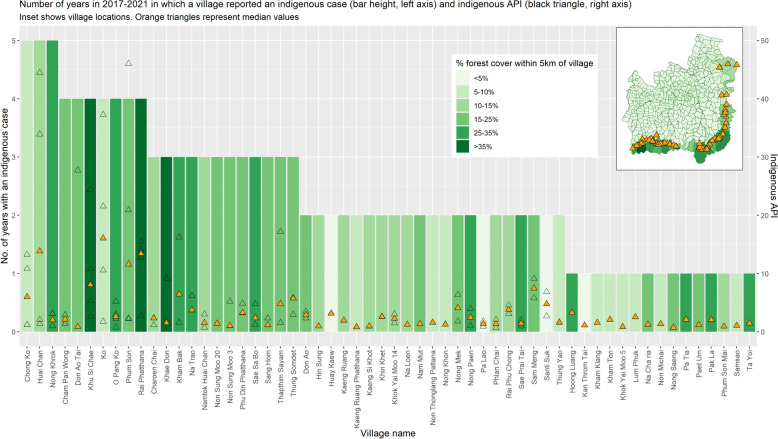
Consistency of indigenous case reporting; indigenous API; and forest cover within a 5 km radius. API: Annual Parasite index

Since 2017, malaria surveillance has been performed in Thailand using the 1-3-7 system [21]. Figure 6 shows the individual villages that have reported indigenous cases since 2017 as well as the raw (black) and median (orange) values for indigenous API in those villages. The colour of the bars indicates forest cover within a 5 km radius. Since 2017, only villages with greater than 5% forest cover within a 5 km radius have reported indigenous cases in more than 2 of the 5 years. Of the 22 villages reporting in 3–5 of the past 5 years, only one had less than 10% forest cover within 5 km. The majority of these villages are located in the highly forested border subdistricts. There was also an association between the proportion of recent years in which a village has reported an indigenous case and the maximum indigenous API. APIs greater than 5 were only reported in villages which had reported cases in 2 or more of the previous 5 years, and APIs greater than 20 (or median APIs greater than 10) were only reported in villages which had cases in at least 4 of the past 5 years. Some villages which consistently reported cases reported low APIs; but high APIs were not seen in villages which did not consistently report cases.

### Estimated R_c_

**Fig. 7:**
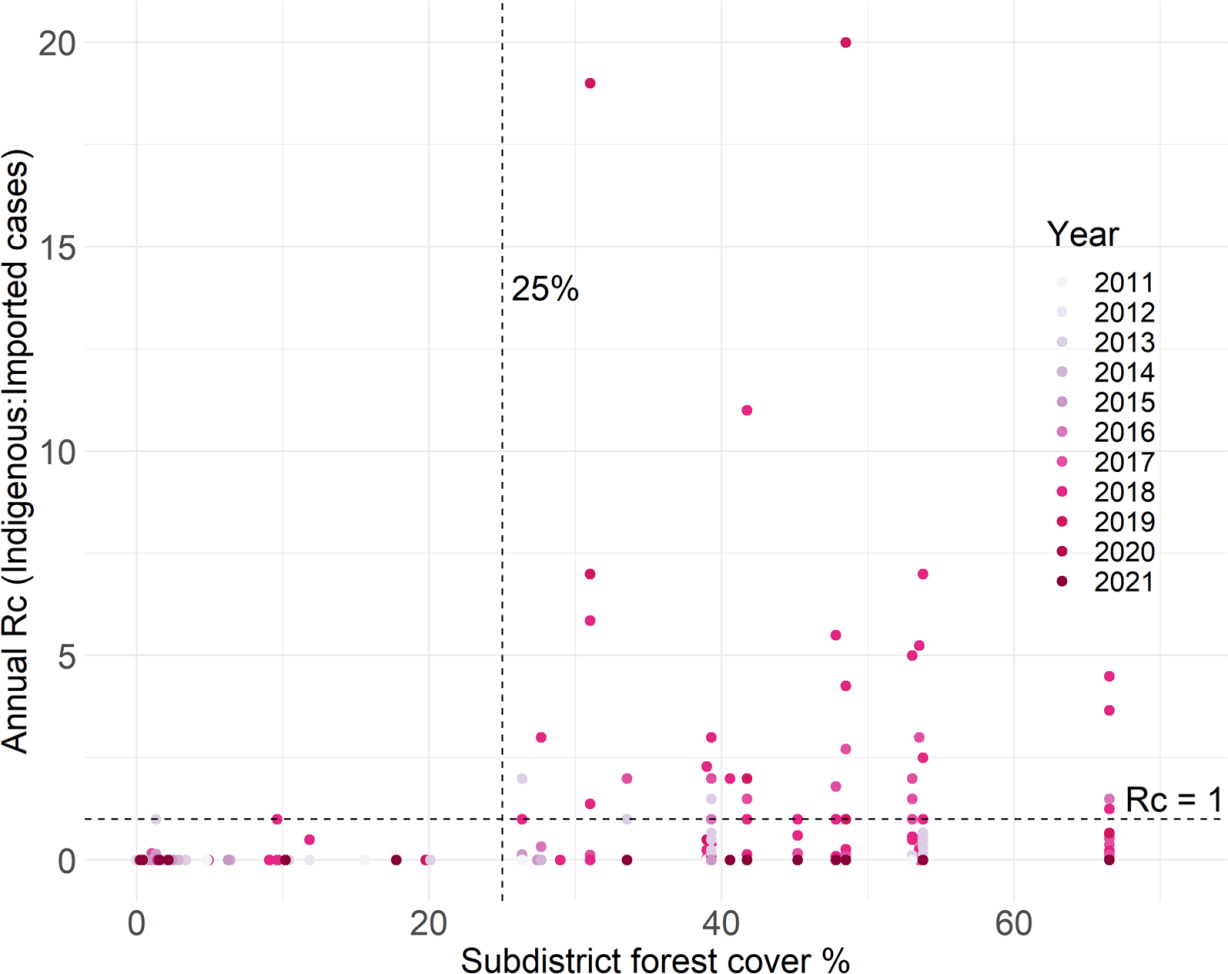
Reproductive number under control (Rc) and subdistrict percentage forest cover

**Fig. 8:**
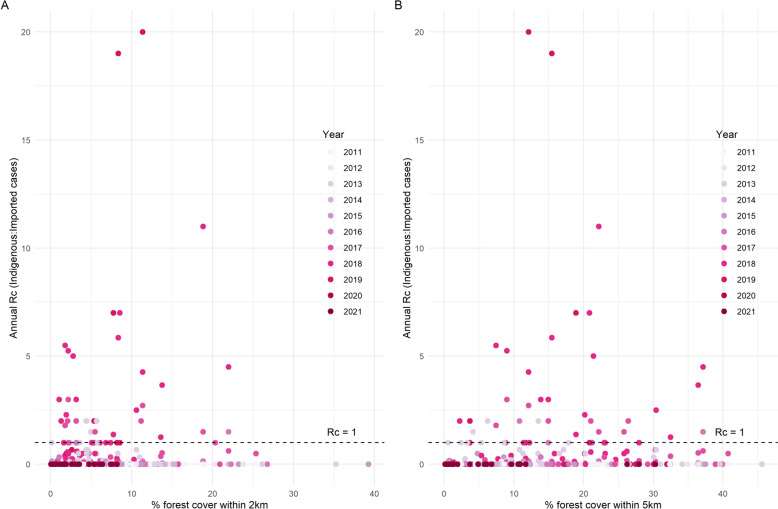
Rc and forest cover within **A** 2 km and **B** 5 km of the village. Rc: Reproductive number under control

Over the study period, an R_c_ greater than 1 was not reported in any village in a subdistrict with less than 25% forest cover (Fig. 7), indicating that endemicity would not be established. As when using API values, the association with percentage forest cover was less clear within a 2 or 5 km radius of the village (Fig. 8). An R_c_ > 1 was not reported in any village with less than 2% forest cover within 5 km, but the majority of villages had greater forest cover than this. There were weakly positive correlations between R_c_ and subdistrict forest cover, and % forest cover within a 5 km radius (Table 1; rho = 0.228 and 0.271, respectively).

### Village risk stratification

**Fig. 9:**
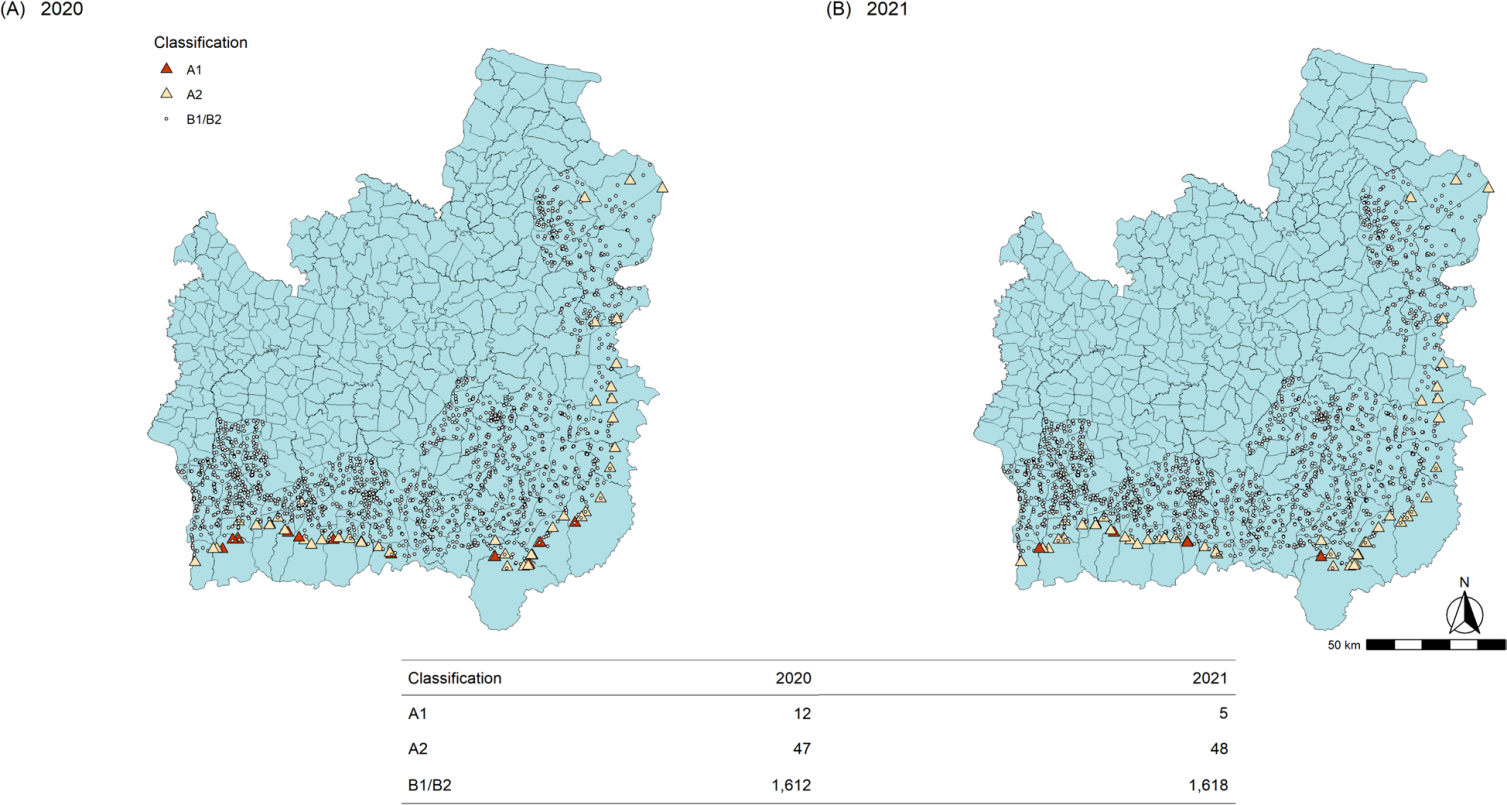
Villages with their risk classifications in **A** 2020 and **B** 2021 per current DVBD criteria in Ubon Ratchathani and Si Sa Ket provinces

No villages which would have been classed as B1 or B2 in 2020 or 2021 (i.e. which had not reported an indigenous case within the last 3 years) reported indigenous transmission in 2020 or 2021. The only villages which reported an indigenous malaria case were A1 or A2 foci, which had reported a case within the previous 3 years. Twelve villages reported indigenous cases in 2020, and five in 2021 (Fig. 9). There were 46 villages classed as A1 or A2 foci in 2020 which did not report an indigenous malaria case in the following year. Almost all A1 and A2 villages were located in the highly forested border subdistricts.

**Fig. 10:**
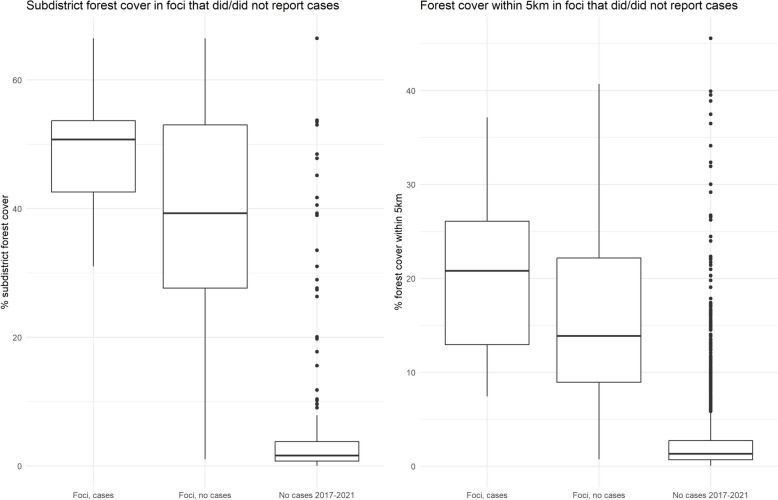
Subdistrict forest cover in villages with/without cases in 2017–2021. Foci, cases = A1/A2 foci in 2017–2019 with subsequent cases in 2020/2021; Foci, no cases = A1/A2 foci in 2017–2019 without subsequent cases in 2020/2021; No cases 2017–2021 = villages with no cases across 2017–2021

The median percentage subdistrict forest cover was higher (50.7%) in A1/A2 foci that reported subsequent cases than in foci that did not (39.3%), although the interquartile ranges overlap (Fig. 10). Both had median values much higher than villages with no recent indigenous cases (1.62%), although there were many outliers. A similar pattern was observed for forest cover within a 5 km radius, whereby the median percentage was higher for foci with cases (20.8%) than without subsequent cases (13.9%) with overlap, and both were greater than the median for villages with no recent cases (1.32%).

**Fig. 11:**
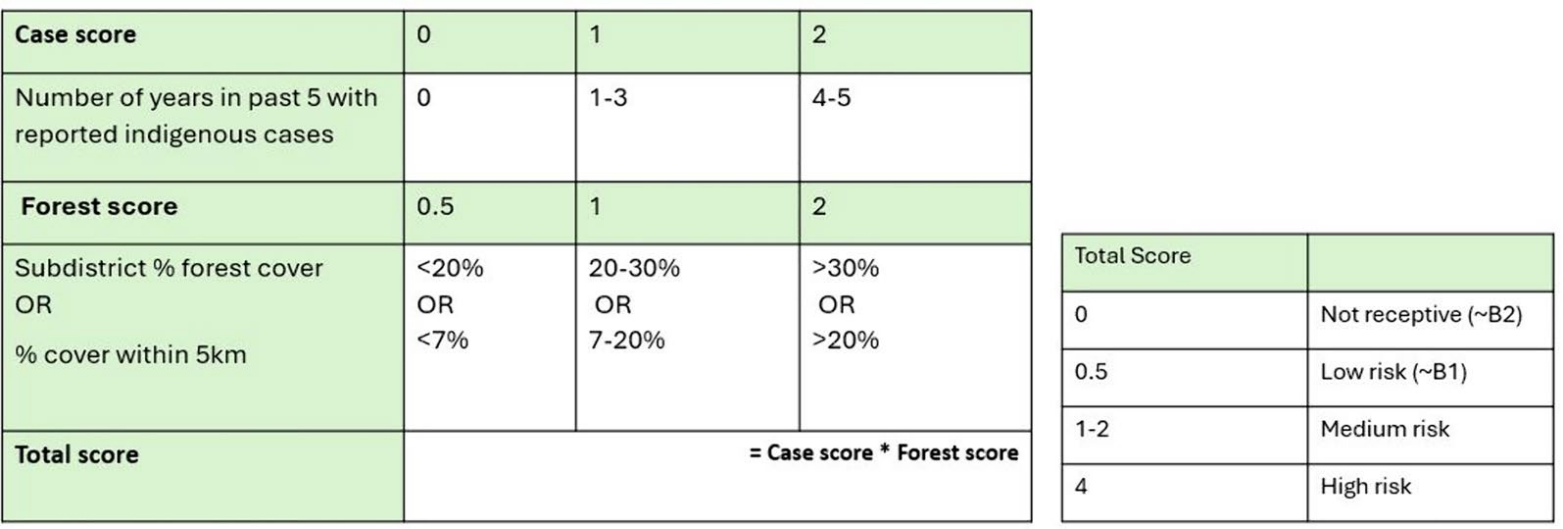
Proposed village risk classification system, utilizing recent malaria case data and forest cover variables

**Fig. 12:**
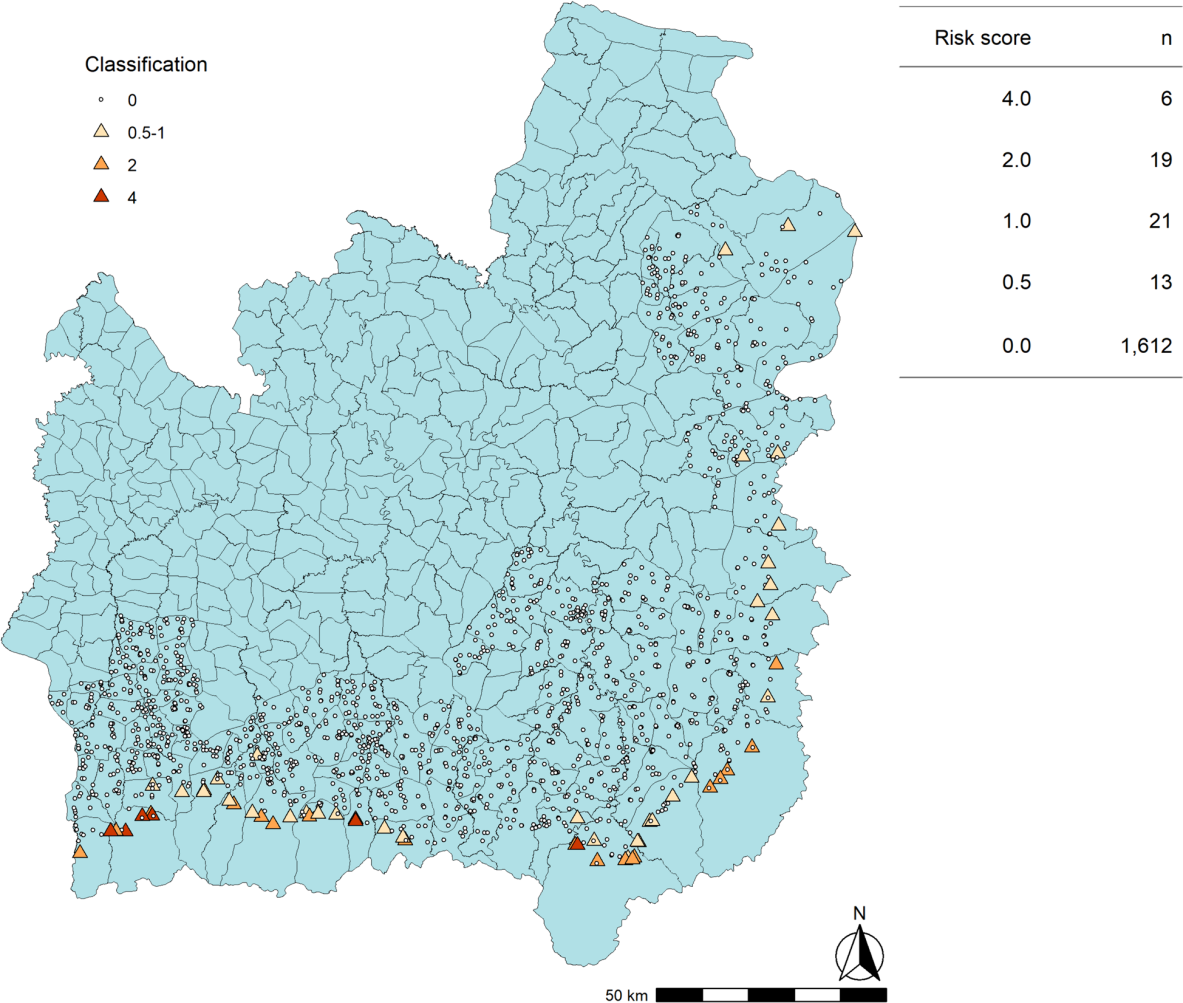
Map of the villages with their risk classifications per the proposed scoring system in Ubon Ratchathani and Si Sa Ket provinces

Based on the results of this analysis, a receptivity classification score was developed (Fig. 11) and compared to the current foci classification system (Fig. 1), which was applied to the case dataset. As A1/A2 status is currently determined by a recent history of indigenous cases, it would be expected to categorize the high-risk villages in a similar way. The numbers and locations of villages with different risk scores are shown in Fig. 12: there were six high risk (score = 4); 40 medium risk (score = 1–2); and 13 lower risk (score = 0.5) villages. Almost all were in the forested border subdistricts. When compared to Fig. 9, which shows the villages categorized per the current risk classification system as above, the spatial distribution of villages was similar. The high-risk villages were the same as the five A1 villages in 2021, plus an extra village which was A1 in 2020. However, there were fewer (40) medium risk villages than A2 villages (48). There were 13 low risk villages, and the rest (1,612) were considered non-receptive. This is the same as the total number of B1/B2 villages in 2020, and fewer than the 1,618 B1/B2 villages in 2021. It was not possible to compare to B1 village numbers alone as this category is based on vector presence, not historical case data. The classification of a village per the DVBD was recorded in the surveillance data with confirmed malaria cases, but comparison was not made to these due to inconsistencies between datasets in the classifications of villages within the same year.

## Discussion

Malaria transmission has a significant environmental component, resulting in spatial heterogeneity in transmission risk and complex epidemiology [16]. Here it was attempted to quantify part of that heterogeneity in a useful and reproducible way. In Si Sa Ket and Ubon Ratchathani provinces in northeastern Thailand, the number of annual cases has declined by 96.6% between 2011 and 2021. There were only 36 cases across the two provinces in 2021. During the study period, in 2017, the Thai DVBD launched its malaria elimination strategy to be used at the local administrative level. This strategy recommended using recent history of indigenous transmission and, in those villages with no recent transmission, vector surveys, to determine receptivity to malaria. Vector surveys are, however, resource-intensive, and results can depend on entomological expertise [4, 10]. The completeness of the surveillance data collected in Thailand has been improving since 2017 [22]. Here a combination of this high-quality surveillance data and forest cover was used as a proxy for receptivity and propose a risk framework which has the potential to facilitate the rationalization of resources targeting malaria in this part of Thailand. This also has scope to be adapted for use in other countries with similar transmission patterns.

In the dataset, indigenous village API was moderately positively associated with metrics of forest cover. Forested areas are conducive to malaria transmission due to the reproduction of vector species in ideal conditions of vegetation cover; temperature; rainfall; humidity; and a lack of infrastructure [16]. The API for cases indigenous to a village was moderately positively correlated with subdistrict percentage forest cover and had lower correlation with the percentage forest cover within a 5 km radius of the village. There were many villages with no reported cases in the past 5 years with high forest cover, which may be receptive but likely have reported no indigenous cases due to low overall case numbers and lack of parasite importation. The R number under control (R_c_) can be approximated as the ratio of indigenous to imported cases and has been used as a proxy for receptivity in previous studies [6–8, 26]. It is generally understood that endemicity will not be established in areas with an R_c_ of less than 1. In the data, only villages located in subdistricts with greater than 25% average forest cover ever had an annual R_c_ greater than 1. The R_c_ has been less than 1 in all villages since 2019, suggesting that progress is being made at a local level towards the goal of elimination in this area.

The greater association of indigenous malaria cases with coarser measures of forest cover such as subdistrict cover, and cover within a 5 km radius, is likely to be multifactorial. There is significant heterogeneity in local geography; environmental factors; and behaviour. Known risk factors for malaria foci in Thailand include the presence of tropical forest and plantations; proximity to international borders; and percentage of short-term residents [2]. The forest forms a natural border between Thailand and Cambodia/Lao PDR, and border subdistricts have the greatest forest cover in the study area. These areas are vulnerable to malaria importation from both forest-going activities and human mobility around border areas, which can introduce the parasite to receptive areas. It is also possible that 1–2 km, typically taken as the flight radius of mosquitos from their breeding sites [30, 31], is too fine a scale to capture the layout of a village. The calculated buffer zones around villages were based on the co-ordinates of a single identifiable site in the village (e.g. a village sign or office) which is not necessarily at the geographic centre, such that the smaller buffers may not fully encompass the village boundaries; nor are villages often laid out in perfect circles. The strength of rank correlation with API was lower when smaller buffer zones were used, and this may be a product of heterogeneity in village layout and location of larval sites.

### Risk classification

As Thailand approaches prevention of re-establishment planning, the focus of malaria strategy shifts to ongoing surveillance and response targeted to areas with high malariogenic potential. The highest risk villages under the current risk classification system (those that reported cases consistently each year) had higher average forest cover metrics.

When considering B1 and B2 foci, it is challenging to identify those that are still receptive to malaria using case data as, by definition, they have not had any recent cases. There were no B1/B2 villages (i.e. those which had not reported any cases in 2017–2019) which subsequently reported an indigenous malaria case in 2020 or 2021. While the best indicator of receptivity to malaria is recent case numbers, this lack of recurrence is representative of the current low-burden situation of the local near-elimination setting. The WHO framework for malaria elimination advises that “In practice, in some settings, non-receptive areas are identified as those that have had no vector control and no locally transmitted malaria cases but have had high-quality surveillance for several years…” [3]. Due to the high-quality surveillance system in place in Thailand, this would apply to many foci, but doesn’t account for areas that may still be receptive but have not had reported cases due to lack of importation. Here it is useful to consider historical case data and its associations with environmental variables, such as forest cover, in order to assess which areas would have cases if the parasite were to be introduced. Combining this with measures of importation known to be associated with probability of reporting indigenous cases, such as proportion of short-term residents [2], would allow further stratification of areas by risk. There was also greater forest cover surrounding the A1/A2 foci which reported subsequent cases than those foci which did not. On the background of local ongoing reduction of malaria cases, this persistence of foci only in the most forested areas may be due to their higher receptivity and vulnerability. The forest cover is greatest on the border, where there is likely a higher risk of malaria importation from reservoirs both in the forest and across the border.

The proposed risk classification tool (Fig. 11) gave a comparable distribution of high and medium-risk villages to the current classification used by the Thai DVBD. This is to be expected, as both incorporate the number of recent years in which indigenous malaria cases have been reported, although the proposed tool also leverages forest cover metrics. This tool identified 13 low risk villages which approximate to the B1 classification (no recent cases, but vectors are present). It was not possible to compare this to the number of B1 villages under the DVBD system for reasons mentioned previously. There were 1612 villages which had a score of zero, which is comparable to the total number of B1/B2 villages in 2020 and 2021 (1612 and 1618, respectively). The advantage of using this tool over the current approach is that it does not rely on entomological data to determine receptivity. While the absence of malaria vectors in an area can be used to infer that it is not receptive [3], this is based on the assumption that an adequate sample was collected; that sampling covered a sufficient geographical area; and that the vector species can be accurately identified. The Hansen forest dataset, however, has been extensively used and validated in tropical forest settings [20, 32, 33], although it is less accurate for local estimates [27]. Both the current and proposed approaches incorporate the high-quality surveillance data currently collected by the Thai DVBD, although it has greater weight in determining the receptivity of low-risk villages in the proposed tool.

However, it was not possible to validate the new risk score for the re-introduction of malaria to a village with no recent cases, as there were no villages classed as B1/B2 which went on to report an indigenous case in 2020 or 2021. This is likely due to the low case numbers and success of ongoing local elimination efforts. Instead, data from 2022 onwards should be used to validate the proposed risk score and adapt it as appropriate.

There is potential for a validated risk score to be adapted for use in other countries in Southeast Asia, particularly those with similar environments, human processes, and forest-based transmission. For it to be reliable, a robust surveillance system would have to be in place. Forest cover data is readily available but would be improved by on the ground validation of satellite data.

## Strengths and limitations

This study combined high-quality surveillance data over a 10-year period with publicly available forest data to develop a reproducible scoring system which has the potential for adaptation to different local requirements. The MORU team also used manually geo-located co-ordinates for villages without co-ordinates in the DVBD datasets.

There are some important limitations. There was a high level of incompleteness from 2012–2016 prior to the introduction of 1-3-7, with many cases not classified by likely origin. This means that comparisons with later data should be made with caution, as there is a higher level of completeness from 2017–2021. The malaria dataset did not differentiate introduced cases, where someone has been infected locally by a mosquito which was infected by an imported case, from those with no link to imported cases, where the original case was also infected locally. In very low-burden settings this has been achieved using spatiotemporal modelling [6, 8], but this is a more complex task where case numbers are higher, such as during the outbreak years in this setting. At one point during the 2014 outbreak in Ubon Ratchathani there were more than 1000 monthly cases.

In Thailand, there are seven *Anopheles* species known to transmit malaria, which have different environmental optima and geographic distributions [31]. If the prevalence of vector species better adapted to urban environments, such as *Anopheles stephensi*, were to significantly increase, estimates of receptivity based on geographical data would have to change significantly [34]. Similarly, the effects of climate change are likely to alter the boundaries of where vectors can breed [35, 36]. Including other factors such as temperature, humidity and well-collected entomological data may improve dynamic estimates of receptivity as the environment changes.

It was not possible to validate the proposed risk score for the re-introduction of malaria in villages with no recent cases, as there have been no indigenous malaria cases reported in B1 or B2 classified villages since the introduction of the 1-3-7 system in 2017. The data available prior to 2017 is of lower quality and completeness. Instead, an example of how the data available could be used to form a stratification system is provided, which can be validated and refined using future surveillance data.

Lastly, the forest cover variables were calculated assuming only loss in the years since 2001. This is because forest gain is harder to detect due to its gradual nature [33]. Therefore, an area may be deforested and rapidly reforested for agroforestry but would be marked as deforested. Thailand has seen increases in malaria cases in workers on coffee and rubber plantations [2, 16]. Future efforts including validation of satellite forest data with on-the-ground photography of forest cover in at-risk areas may provide better insights into the true forest cover. Other future efforts could also include qualitative studies to explore human factors affecting receptivity and the impact of foci management interventions.

## Conclusions

Thailand's efforts to eliminate malaria by 2024 have been accompanied by a shift in malaria strategy from elimination to prevention of re-establishment in malaria free provinces. The current risk stratification system used by the Thai DVBD involves the most active interventions in villages with recent reported cases, and the collection and identification of vectors in mosquito traps in villages which have not reported indigenous cases in the last 3 years [1]. It was found that the rates of reporting of malaria cases indigenous to a village were more strongly associated with coarse measures of forest cover, such as cover within a 5 km radius and the forest cover within a subdistrict. There was a weak relationship with cover within a 1 or 2 km radius, or the distance to the nearest forested area, which may be a product of human and environmental factors. Leveraging existing high-quality malaria case data and forest cover data to identify the degree to which a certain area is conducive to malaria transmission will likely be more cost and time effective. A village risk stratification system is proposed which requires validation using future malaria case data. This analysis also has the potential to inform strategy for locations with similar transmission patterns and human processes to Thailand, such as in other Southeast Asian countries.

## Data Availability

The population counts per village datasets analysed during the current study are available in the Official statistics registration systems repository, https://stat.bora.dopa.go.th/new_stat/webPage/statByYear.php. The village GPS coordinates collected by our field staff and in Street View in Google Maps are available from the corresponding author on reasonable request. The Thailand administrative boundaries dataset used in the current study is publicly available at https://data.humdata.org/dataset/cod-ab-tha. The malaria surveillance dataset and village GPS coordinates in the DVBD surveillance database analysed during the current study are not publicly available as they belong to the Department of Disease Control, Ministry of Public Health, Thailand, but are available on reasonable request.

## Abbreviations

GMS: Greater Mekong Subregion
POR: Prevention of re-establishment
WHO: World Health Organization
ITN: Insecticide-treated net
IRS: Indoor residual spraying
DVBD: Division of Vector-Borne Disease
GPS: Global Positioning System
RDT: Rapid diagnostic test
API: Annual Parasite Index

## References

[1] Guide to Malaria Elimination for Thailand. https://malaria.ddc.moph.go.th/downloadfiles/Guide%20to%20Malaria%20Elimination%20for%20Thailand%20LAO_EN.pdf. Accessed 6 Jul 2023.

[2] Prempree P, Bisanzio D, Sudathip P, Kanjanasuwan J, Powell I, Gopinath D, et al. Environmental factors linked to reporting of active malaria foci in Thailand. Trop Med Infect Dis. 2023;8:179. 36977180 10.3390/tropicalmed8030179 PMC10051531

[3] WHO. A framework for malaria elimination. Geneva, World Health Organization, 2017. https://www.who.int/publications-detail-redirect/9789241511988. Accessed 26 Jul 2023.

[4] Yukich JO, Lindblade K, Kolaczinski J. Receptivity to malaria: meaning and measurement. Malar J. 2022;21:145. 35527264 10.1186/s12936-022-04155-0 PMC9080212

[5] WHO. Malaria terminology. Geneva, World Health Organization, 2021. https://apps.who.int/iris/bitstream/handle/10665/349442/9789240038400-eng.pdf?sequence=1&isAllowed=y. Accessed 6 Jul 2023.

[6] Reiner RC Jr, Le Menach A, Kunene S, Ntshalintshali N, Hsiang MS, Perkins TA, et al. Mapping residual transmission for malaria elimination. Elife. 2015;4: e09520. 26714110 10.7554/eLife.09520 PMC4744184

[7] Churcher TS, Cohen JM, Novotny J, Ntshalintshali N, Kunene S, Cauchemez S. Measuring the path toward malaria elimination. Science. 2014;344:1230–2. 24926005 10.1126/science.1251449 PMC4340075

[8] Routledge I, Chevéz JER, Cucunubá ZM, Rodriguez MG, Guinovart C, Gustafson KB, et al. Estimating spatiotemporally varying malaria reproduction numbers in a near elimination setting. Nat Commun. 2018;9:2476. 29946060 10.1038/s41467-018-04577-y PMC6018772

[9] Sagna AB, Kibria MG, Naher S, Islam S, Aktaruzzaman MM, Alam MS, et al. Stratifying malaria receptivity in Bangladesh using archived rapid diagnostic tests. Malar J. 2020;19:345. 32967671 10.1186/s12936-020-03418-y PMC7513508

[10] Burkot TR, Bugoro H, Apairamo A, Cooper RD, Echeverry DF, Odabasi D, et al. Spatial-temporal heterogeneity in malaria receptivity is best estimated by vector biting rates in areas nearing elimination. Parasit Vectors. 2018;11:606. 30482239 10.1186/s13071-018-3201-1 PMC6260740

[11] Dye C. Vectorial capacity: must we measure all its components? Parasitol Today. 1986;2:203–9. 15462840 10.1016/0169-4758(86)90082-7

[12] Noor AM, Uusiku P, Kamwi RN, Katokele S, Ntomwa B, Alegana VA, et al. The receptive versus current risks of *Plasmodium falciparum* transmission in Northern Namibia: implications for elimination. BMC Infect Dis. 2013;13:184. 23617955 10.1186/1471-2334-13-184 PMC3639180

[13] Battle KE, Cameron E, Guerra CA, Golding N, Duda KA, Howes RE, et al. Defining the relationship between *Plasmodium vivax* parasite rate and clinical disease. Malar J. 2015;14:191. 25948111 10.1186/s12936-015-0706-3 PMC4429942

[14] Salahi-Moghaddam A, Turki H, Yeryan M, Fuentes MV. Spatio-temporal prediction of the malaria transmission risk in Minab District (Hormozgan Province, Southern Iran). Acta Parasitol. 2022;67:1500–13. 35951221 10.1007/s11686-022-00598-2 PMC9705508

[15] Bueno-Marí R, Jiménez-Peydró R. Study of the malariogenic potential of Eastern Spain. Trop Biomed. 2012;29:39–50. 22543601

[16] Kar NP, Kumar A, Singh OP, Carlton JM, Nanda N. A review of malaria transmission dynamics in forest ecosystems. Parasit Vectors. 2014;7:265. 24912923 10.1186/1756-3305-7-265 PMC4057614

[17] Sawyer DR. Malaria on the Amazon frontier: economic and social aspects of transmission and control. Southeast Asian J Trop Med Public Health. 1986;17:342–5. 3563600

[18] Laporta GZ, Ilacqua RC, Bergo ES, Chaves LSM, Rodovalho SR, Moresco GG, et al. Malaria transmission in landscapes with varying deforestation levels and timelines in the Amazon: a longitudinal spatiotemporal study. Sci Rep. 2021;11:6477. 33742028 10.1038/s41598-021-85890-3 PMC7979798

[19] Jongdeepaisal M, Khonputsa P, Prasert O, Maneenet S, Pongsoipetch K, Jatapai A, et al. Forest malaria and prospects for anti-malarial chemoprophylaxis among forest goers: findings from a qualitative study in Thailand. Malar J. 2022;21:47. 35164759 10.1186/s12936-022-04070-4 PMC8845363

[20] Rerolle F, Dantzer E, Lover AA, Marshall JM, Hongvanthong B, Sturrock HJ, et al. Spatio-temporal associations between deforestation and malaria incidence in Lao PDR. Elife. 2021;10: e56974. 33686939 10.7554/eLife.56974 PMC8024023

[21] Lertpiriyasuwat C, Sudathip P, Kitchakarn S, Areechokchai D, Naowarat S, Shah JA, et al. Implementation and success factors from Thailand’s 1-3-7 surveillance strategy for malaria elimination. Malar J. 2021;20:201. 33906648 10.1186/s12936-021-03740-z PMC8076878

[22] Sudathip P, Naowarat S, Kitchakarn S, Gopinath D, Bisanzio D, Pinyajeerapat N, et al. Assessing Thailand’s 1-3-7 surveillance strategy in accelerating malaria elimination. Malar J. 2022;21:222. 35850687 10.1186/s12936-022-04229-z PMC9294779

[23] Sumarnrote A, Corbel V, Overgaard HJ, Celhay O, Marasri N, Fustec B, et al. Plasmodium infections in *Anopheles* mosquitoes in Ubon Ratchathani province, Northeastern Thailand during a malaria outbreak. J Am Mosq Control Assoc. 2018;34:11–7. 31442122 10.2987/17-6715.1

[24] Chen T, Zhang S, Zhou SS, Wang X, Luo C, Zeng X, et al. Receptivity to malaria in the China-Myanmar border in Yingjiang County, Yunnan Province, China. Malar J. 2017;16:478. 29162093 10.1186/s12936-017-2126-z PMC5699173

[25] Official statistics registration systems. Population Counts. Administration and Registration Technology Development Division, Bureau of Registration Administration, Department of Provincial Administration, Ministry of Interior. https://stat.bora.dopa.go.th/new_stat/webPage/statByYear.php. Accessed 24 Dec 2023.

[26] Cohen JM, Moonen B, Snow RW, Smith DL. How absolute is zero? An evaluation of historical and current definitions of malaria elimination. Malar J. 2010;9:213. 20649972 10.1186/1475-2875-9-213 PMC2983111

[27] Hansen MC, Potapov PV, Moore R, Hancher M, Turubanova SA, Tyukavina A, et al. High-resolution global maps of 21st-Century forest cover change. Science. 2013;342:850–3. 24233722 10.1126/science.1244693

[28] QGIS development team. QGIS Geographic Information System. Open Source Geospatial Foundation; 2009. http://qgis.org

[29] R Core Team. R: a language and environment for statistical computing. Vienna, Austria: R Foundation for Statistical Computing; 2022. https://www.R-project.org/

[30] Kaufmann C, Briegel H. Flight performance of the malaria vectors *Anopheles gambiae* and *Anopheles atroparvus*. J Vector Ecol. 2004;29:140–53. 15266751

[31] Tsuda Y, Suwonkerd W, Takagi M. Mark-release-recapture studies on flight distance and survival rate of anopheline mosquitoes (Diptera: Culicidae) in Northern Thailand. Med Entomol Zool. 2011;62:85–92. 10.7601/mez.62.85

[32] Hansen A, Barnett K, Jantz P, Phillips L, Goetz SJ, Hansen M, et al. Global humid tropics forest structural condition and forest structural integrity maps. Sci Data. 2019;6:232. 31653863 10.1038/s41597-019-0214-3 PMC6814722

[33] Potapov P, Hansen MC, Pickens A, Hernandez-Serna A, Tyukavina A, Turubanova S, et al. The global 2000–2020 land cover and land use change dataset derived from the Landsat archive: first results. Front Remote Sens. 2022. 10.3389/frsen.2022.856903. 10.3389/frsen.2022.856903

[34] WHO. Vector alert: *Anopheles stephensi* invasion and spread in Africa and Sri Lanka. Geneva, World Health Organization, 2023. https://www.who.int/publications-detail-redirect/9789240067714. Accessed 3 Aug 2023.

[35] Patz JA, Olson SH. Malaria risk and temperature: influences from global climate change and local land use practices. Proc Natl Acad Sci USA. 2006;103:5635–6. 16595623 10.1073/pnas.0601493103 PMC1458623

[36] Fouque F, Reeder JC. Impact of past and on-going changes on climate and weather on vector-borne diseases transmission: a look at the evidence. Infect Dis Poverty. 2019;8:51. 31196187 10.1186/s40249-019-0565-1 PMC6567422

